# Asexuality Disclosure in Healthcare: Attachment and Patient‐Reported Experiences in a Cross‐Sectional Pilot Survey

**DOI:** 10.1002/hsr2.72094

**Published:** 2026-03-11

**Authors:** Kyung‐Eun (Anna) Choi, Rosa Aschenbrenner, Sebastian Fitzek

**Affiliations:** ^1^ Health Services Research Group, Medical Image Analysis & AI Danube Private University, Krems an der Donau Austria; ^2^ Center for Health Services Research Brandenburg Medical School (MHB) Neuruppin Germany; ^3^ Evidence‐Based Practice in Brandenburg — A JBI Affiliated Group, Brandenburg an der Havel, Germany University of Adelaide Australia; ^4^ Student of Human Medicine Danube Private University, Krems an der Donau Austria

**Keywords:** allonormativity, asexuality, attachment, disclosure, healthcare, patient‐reported outcomes

## Abstract

**Background and Aims:**

Asexual adults often report misunderstandings during clinical encounters. We described romantic attachment (ECR‐R anxiety/avoidance) in an asexual sample and tested whether disclosure in medical settings was associated with feelings of misunderstanding, stigma, and a (dis) comfort‐discussing orientation.

**Methods:**

This was a cross‐sectional online pilot survey (*N* = 47, aged 18–67 years). Variables: Disclosure (no/never, depends/sometimes, yes/always), Likert outcomes (1–5), ECR‐R (1–7), and age. Analyses: ordinary least squares (OLS) with HC3 robust SEs; ordered logit sensitivity analyses (odds ratios, 95% CI) in Supplement S1; multiplicity controlled via Benjamini–Hochberg FDR.

**Results:**

Mean ECR‐R scores: anxiety 3.28 (SD 1.30), avoidance 3.47 (SD 1.41). Avoidance was higher in sex‐averse than in sex‐favourable participants (Hedges' *g* = 1.285; 95% CI 0.449–2.769). Relative to never disclosing, “depends/sometimes” was associated with greater misunderstanding (OR 5.69; 95% CI 1.45–22.32) and stigma (OR 5.32; 1.30–21.80), whereas “yes/always” was associated with less discomfort (OR 0.05; 0.00–0.68). Estimates for “always” disclosure reflect a small subgroup (*n* = 4). Key contrasts were robust to FDR adjustment, and ordinal models were directionally consistent.

**Conclusion:**

Disclosure patterns are related to reported healthcare experiences of asexual patients. Non‐presumptive sexual history taking, patient‐led disclosure options (e.g., an “asexual” field), and asexuality‐competent communication training may improve care. Larger multi‐site studies are warranted.

## 1 Introduction

Asexuality is best understood as a spectrum and umbrella term rather than a unitary absence of sexual attraction. Contemporary accounts highlight the split‐attraction model, which distinguishes sexual from romantic attraction and helps to characterise heterogeneity across ace‐spectrum identities [1, 2]. In healthcare, routine sexual history‐taking can inadvertently assume sexual desire or activity, which risks misunderstanding and pathologising responses when asexual patients disclose [3, 4, 5]. We use the term ‘asexual spectrum (ace)’ to refer to this umbrella, and subsequently use ‘ace’ or ‘asexual spectrum’ for brevity.

We explicitly name allonormativity—the taken‐for‐granted assumption that sexual desire and activity are universal—as a root driver of anti‐asexual stigma in healthcare. Allonormativity helps explain why disclosures may be met with misunderstanding or pathologising responses and is documented in recent health‐communication work with asexual people of colour [6].

Empirical work suggests that clinician knowledge gaps and allonormative assumptions can shape asexual patients' experiences in care, including disbelief, inappropriate pathologising language, and barriers to inclusive services [3, 4, 5, 6, 7, 8]. Attachment, assessed with the Experiences in Close Relationships–Revised (ECR‐R) scale, offers a useful lens for communication and trust; normative online resources enable contextual interpretation of scores [9, 10].

A rapidly growing body of scholarship now details (a) empirical patterns across asexual populations, (b) clinical communication pitfalls, and (c) concrete guidance for affirming practices. A scoping review maps the expansion of social science evidence on asexuality and identifies persistent clinical knowledge gaps [7]. Practice‐oriented syntheses outline asexual‐competent care—neutral sexual history taking, non‐pathologising language, and explicit intake options for “asexual” and microlabels [4, 5]. Qualitative guidance for clinicians similarly reframes “sexual disinterest” outside deficit models and emphasises validation during disclosure [8]. Patient reports from healthcare settings range from explicitly aggressive to affirming interactions, underscoring how clinician stance shapes experiences [11]. Collectively, these strands position our pilot as directly relevant to ongoing efforts to reduce allonormative drift in routine care.

This pilot study addressed two questions: (1) What are the ECR‐R anxiety/avoidance levels in an asexual sample and do they differ within the group by attitude toward sex (averse/indifferent/favourable)? (2) Is the disclosure of asexuality in healthcare associated with misunderstanding, stigma, and (dis)comfort discussing orientation after accounting for age and attachment? Reporting follows STROBE [12], and patient and public involvement (PPI) is described per GRIPP2‐SF [13].

Why is attachment clinically relevant? Romantic attachment (ECR‐R) is relevant because it shapes expectations for trust, disclosure, and pacing in patient–provider interactions. Therefore, we situate attachment as contextual, not diagnostic, recognising that higher avoidant scores may reflect valid relational preferences rather than deficits. We also note that ECR‐R targets romantic relationships, and interpretations do not extend to friendships or other non‐romantic ties.

## 2 Methods

### 2.1 Measures

We assessed adult romantic attachment using the Experiences in Close Relationships–Revised (ECR‐R) scale, computing anxiety and avoidance as participant‐level means for the respective items (1–7). The instrument targets romantic relationships and is used here to contextualise communication and trust in care; normative resources were consulted only for context [9, 10].

Disclosure was measured with: “Do you disclose your asexuality in a medical setting?” (response options: no/never, depends/sometimes, yes/always).

Patient‐reported experiences (1–5; higher = more of the attribute) included feeling misunderstood because of asexuality, discrimination/stigma, and (dis)comfort discussing sexual orientation with providers as well as perceived inclusivity of care and ease of use of general and mental health services.

To improve reliability and interpretability, we constructed two composites: a health literacy index (finding, accessing, evaluating, using health information; items were *z*‐scored and averaged; α = 0.88) and a misunderstanding/stigma index (misunderstanding, stigma, and perceived effects on quality; items were *z*‐scored and averaged; α = 0.90).

The covariates were age (years) and the two ECR‐R dimensions, attitudes toward sex (averse/indifferent/favourable), which were used for within‐group contrasts.

Setting up online recruitment via asexual community platforms and university channels in Europe. Data were collected from March to May 2025.

Participants Adults (≥ 18 years old), self‐identified as asexual. Of the 49 respondents with attachment data, 47 provided usable responses (analytical sample).

Bias Self‐selection and self‐reporting may introduce bias, and the cross‐sectional design precludes causal inference.

Study size Pragmatic for pilot; no formal power calculation.

### 2.2 Statistical Analysis

We summarised the sample and contrasted the ECR‐R between sex‐attitude groups using Hedges' g with bootstrap 95% CIs (10,000 draws). For 5‐point outcomes, we fitted ordinary least squares (OLS) models with HC3 robust standard errors, regressing each outcome on disclosure (reference = no/never), age, ECR‐R anxiety, and ECR‐R avoidance. Ordered logistic regressions served as sensitivity analyses and are reported as odds ratios (95% CI) in Supplement S1. To control multiplicity across key coefficients, we applied the Benjamini–Hochberg false discovery rate (FDR) [14]. Missingness was modest; the analyses used listwise deletion per model (no imputation). All tests were two‐sided with α = 0.05. Analyses were conducted in Python 3.11 (pandas 2.2.2; numpy 1.26.4; statsmodels 0.14.2; scipy 1.13.1). We followed the SAMPL where applicable.

### 2.3 Patient and Public Involvement

Community members advised recruitment wording, piloted the questionnaire for clarity (microlabels and clinical scenarios), and reviewed the lay‐summary plan. They did not participate in data analysis or authorship decisions. A complete GRIPP2‐SF checklist is provided in the Supplement [13].

### 2.4 Ethical Approval

This study was approved by the Danube Private University Ethics Committee (DPU‐EK/095) and complied with the Declaration of Helsinki. Participation was anonymous and voluntary and all participants provided informed consent.

## 3 Results

### 3.1 Sample Characteristics

**Figure 1:**
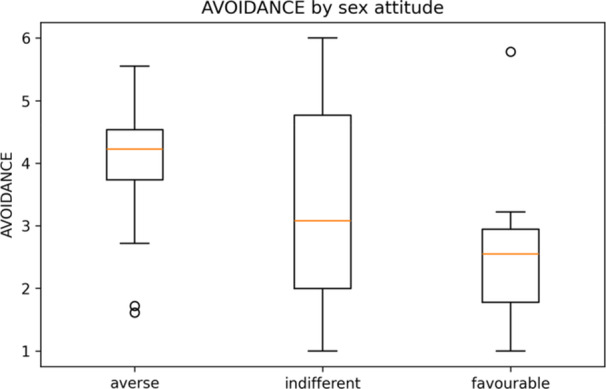
ECR‐R avoidance by attitude toward sex (averse/indifferent/favourable). *Note:* Boxplots show medians (horizontal line), interquartile range (box), and whiskers to 1.5×IQR; circles denote outliers. The score was ECR‐R avoidance (1–7). Descriptive statistics (no covariate adjustment). See Table 2 for the between‐group contrasts.

**Figure 2:**
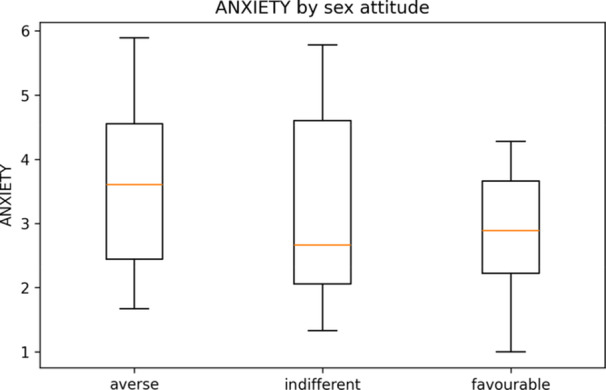
ECR‐R Anxiety by attitude toward sex (averse/indifferent/favourable). *Note:* Boxplots show medians (horizontal line), interquartile range (box), and whiskers to 1.5×IQR; circles denote outliers. The score was ECR‐R anxiety (1–7). Descriptive statistics (no covariate adjustment). See Table 2 for the between‐group contrasts.

**Figure 3:**
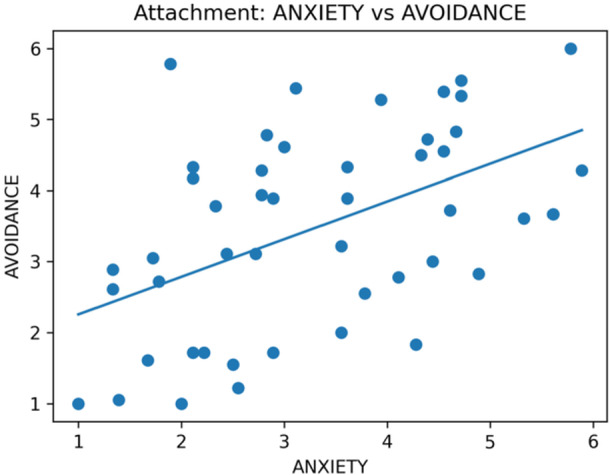
Scatterplot of anxiety versus avoidance with a least‐squares line. *Note:* Each point represents one participant. The line shows the least‐squares fit (no covariate adjustment). Axes show ECR‐R subscale scores (1–7; observed range 1.0–6.0). This figure does not include the disclosure categories. See Table 2 for between‐group contrasts and Table 3/Supplement S1 for modelled estimates.

There were 47/49 usable responses (mean age 30.8 ± 11.6 years). Gender identity: 29/47 women (62%), 10/47 men (21%), and 8/47 non‐binary/other (17%). Attitude toward sex: 22 averse, 14 indifferent, and 11 favourable. The mean ECR‐R scores were 3.28 for anxiety and 3.47 for avoidance (Table 1). The disclosure distribution was: no/never 16, depends/sometimes 27, yes/always 4; the “yes/always” subgroup was n = 4 (Table 1). Distributions are shown in Figure 1 (avoidance) and Figure 2 (anxiety), and their associations are shown in Figure 3 (equivalent to 34.0%, 57.4%, and 8.5% of the sample).

**Table 1:** Participant characteristics and descriptive statistics.

Metric	Value
*N* (usable)	47
Age (mean)	30.83
Age (SD)	11.56
Age (min–max)	18–67
Attitude: averse	22
Attitude: indifferent	14
Attitude: favourable	11
Disclosure: depends	27
Disclosure: no	16
Disclosure: yes	4
ANXIETY mean	3.28
ANXIETY SD	1.31
ANXIETY min–max	1.0–5.89
AVOIDANCE mean	3.47
AVOIDANCE SD	1.41
AVOIDANCE min–max	1.0–6.0
Health Literacy α	0.881
Misunderstanding/Stigma α	0.901

*Note:* The ECR‐R subscales range from 1 to 7 (higher=higher traits). The outcome items ranged from 1 to 5, unless otherwise noted. α=Cronbach's alpha for composite indices.

### 3.2 Attachment Profiles and Within‐Group Contrasts

The between‐group contrasts in ECR‐R are summarised in Table 2. Avoidance was higher in sex‐averse than in sex‐favourable participants (Hedges' *g* = 1.285; 95% CI 0.449–2.769).

**Table 2:** Within‐group contrasts in ECR‐R by attitude toward sex (Hedges' *g* with bootstrap 95% CIs).

Contrast	Variable	Hedges *g*	95% CI	*n*1	*n*2
averse ‐ favourable	AVOIDANCE	1.285	[0.449, 2.769]	22	11
averse ‐ indifferent	AVOIDANCE	0.517	[−0.191, 1.391]	22	14
averse ‐ favourable	ANXIETY	0.538	[−0.136, 1.265]	22	11
averse ‐ indifferent	ANXIETY	0.238	[−0.450, 0.993]	22	14

*Note:* ECR‐R = Experiences in Close Relationships–Revised. Hedges’ *g* was computed using 10,000 bootstrap resamples to derive 95% confidence intervals. n1 and n2 denote the group sizes.

### 3.3 Disclosure and Patient‐Reported Experiences

**Figure 4:**
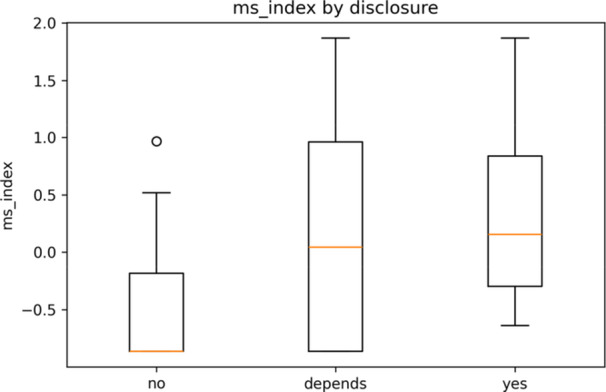
Misunderstanding/Stigma Index by disclosure (no/depends/yes). *Note:* Box plots are shown above. The index is the *z*‐scored mean of misunderstanding, stigma, and the perceived effect on care quality (higher = more misunderstanding/stigma). Descriptive statistics (no covariate adjustment). See Table 3 and Supplement S1 for the model estimates and odds ratios.

**Figure 5:**
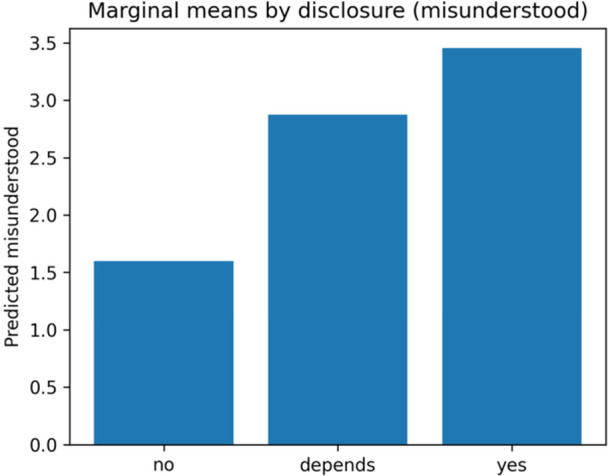
Adjusted marginal means for misunderstanding by disclosure (covariates: age, anxiety, and avoidance). *Note:* Bars show OLS model‐predicted means with HC3 robust SEs, adjusting for age, ECR‐R anxiety, and ECR‐R avoidance. The reference disclosure category in Table 3 is “no/never.” No error bars are plotted here; see Table 3 for the 95% CIs and *q*(FDR) values. Supplement S1 reports ordered‐logit odds ratios.

Unadjusted outcome distributions by disclosure are shown in Figure 4, and adjusted marginal means are shown in Figure 5. Table 3 presents the full regression estimates. Relative to no/never disclosing, “depends/sometimes” was associated with greater misunderstanding (β = 1.28; 95% CI 0.48–2.08; *p* = 0.002; *q* = 0.011) and stigma (β = 1.19; 95% CI 0.37–2.01; *p* = 0.004; *q* = 0.017). The “yes/always” category was associated with less discomfort discussing orientation (β = −1.67; 95% CI − 2.53 to −0.81; *p* < 0.001; *q* = 0.002). Ordered logit sensitivity analyses were directionally consistent and are reported as odds ratios in Supplement S1 (e.g. misunderstanding OR 5.69, 95% CI 1.45–22.32; stigma OR 5.32, 95% CI 1.30–21.80; discomfort OR 0.05, 95% CI 0.00–0.68).

**Table 3:** Associations between disclosure and patient‐reported outcomes (OLS with HC3, FDR‐adjusted).

outcome	term	β (95% CI)	*p*	q(FDR)
misunderstood	ANXIETY	0.34 (−0.05, 0.74)	0.09	0.210
misunderstood	AVOIDANCE	0.09 (−0.30, 0.48)	0.66	0.787
misunderstood	C(disclose)[T. depends]†	1.28 (0.48, 2.08)	0.002	0.011
misunderstood	C(disclose)[T. yes]	1.85 (−0.18, 3.89)	0.07	0.210
stigma	ANXIETY	0.22 (−0.20, 0.65)	0.30	0.531
stigma	AVOIDANCE	0.10 (−0.33, 0.53)	0.64	0.787
stigma	C(disclose)[T. depends]†	1.19 (0.37, 2.01)	0.004	0.017
stigma	C(disclose)[T. yes]	1.07 (−1.75, 3.90)	0.46	0.685
uncomfortable	ANXIETY	−0.05 (−0.44, 0.33)	0.79	0.864
uncomfortable	AVOIDANCE	0.16 (−0.15, 0.46)	0.31	0.531
uncomfortable	C(disclose)[T. depends]	−0.02 (−0.92, 0.88)	0.96	0.962
uncomfortable	C(disclose)[T. yes]†	−1.67 (−2.53, −0.81)	< 0.001	0.002

*Note:* Models adjusted for age, ECR‐R anxiety, and ECR‐R avoidance; reference for disclosure = no/never. The 95% CIs were computed as β ± 1.96×SE_HC3. The terms marked † remain significant at q(FDR) < 0.05.

Table 3 presents full regression estimates.

## 4 Discussion

### 4.1 Principal Findings

Participants showed higher scores on the avoidant dimension of the ECR‐R; we interpreted this as a relational preference pattern rather than a deficit. Disclosure patterns related to patient‐reported experiences: selective disclosure (“depends”) aligned with more misunderstanding/stigma, whereas consistent disclosure (“yes/always”) aligned with less discomfort in discussing orientation. We note the small size of the “yes/always” subgroup (*n* = 4), which widens the confidence intervals and warrants cautious interpretation.

### 4.2 Interpretation and Implications

These patterns are intelligible within the lens of allonormativity, which normalises sexual desire/activity as universal and renders ace disclosures susceptible to disbelief or medicalising framing [6]. Clinically, reducing allonormative drift in sexual history‐taking (neutral prompts; explicit inclusion of “asexual” on forms) aligns with emerging recommendations for ace‐affirming care [4, 5]. Positive encounters are typically tied to clinicians familiar with asexuality and non‐pathologising responses to disclosure; negative encounters often involve disbelief or medicalising framings [3, 11]. We used the ECR‐R to contextualise communication and trust in clinical encounters rather than to diagnose, because ECR‐R targets romantic relationships, and interpretations do not extend to non‐romantic ties. The recommended communication practices closely map these findings, using neutral prompts and affirming language and non‐presumptive sexual history taking, explicit “asexual” options on forms, and brief clinician training in asexual‐affirming microskills [4, 5, 8].

### 4.3 Strengths and Limitations

Strengths include a clearly defined asexual sample, validated attachment measures, transparent effect‐size reporting with HC3 robust SEs, sensitivity analyses (ordered logit), control of multiplicity via FDR, and PPI elements. Limitations include small convenience sampling (generalisability), self‐report measures, cross‐sectional design (no causal inference), and lack of a matched allosexual comparison group. The small “yes/always” subgroup (*n* = 4) limits precision; item‐level ECR‐R data were unavailable for internal‐consistency estimates; clinical context (e.g., specialty) was not captured in detail. Analyses of the perceived inclusivity of care and ease of use were exploratory.

### 4.4 Future Research

Larger, multisite studies should test clinician‐facing interventions (inclusive intake forms and brief microskill training) on patient‐reported outcomes, incorporate observational or audio‐recorded visits, and examine intersectional effects (e.g. gender identity, aromantic identity, and trauma history) with adequate power.

## 5 Conclusions

Among adults identified as asexual, avoidance scores were higher within the sample, and disclosure patterns were associated with more misunderstanding/stigma (for selective disclosure) but less discomfort in discussing orientation (for consistent disclosure). Clinicians can improve the patient experience through non‐assumptive, asexuality‐competent, and patient‐led disclosure options.

### 5.1 Implications for Practice

- Ask, do not assume: use neutral prompts that do not presuppose sexual attraction or activity.
- Offer low‐burden disclosure: include “asexual” (and microlabels where appropriate) on forms; display inclusive signage.
- Acknowledge and normalise: if a patient discloses asexuality, validate and clarify without pathologising.
- Pace shared decision‐making: If avoidant tendencies are present, pace information‐giving and choice‐offering.

## Author Contributions

Conceptualisation: Kyung‐Eun Choi, Sebastian Fitzek. Methodology: Kyung‐Eun Choi, Sebastian Fitzek. Software: Sebastian Fitzek. Validation: Rosa Aschenbrenner, Sebastian Fitzek. Formal Analysis: Sebastian Fitzek. Investigation: Rosa Aschenbrenner. Data Curation: Rosa Aschenbrenner. Writing – Original Draft: Sebastian Fitzek. Writing – Review and Editing: Kyung‐Eun Choi, Rosa Aschenbrenner, Sebastian Fitzek. Visualization: Sebastian Fitzek. Supervision: Kyung‐Eun Choi, Sebastian Fitzek. Project Administration: Sebastian Fitzek.

## Funding

This research received no specific grant. The funder(s) had no role in the study design, data collection, analysis, interpretation, writing of the report, or the decision to submit the manuscript for publication.

## Ethics Statement

DPU‐EK/095 (Danube Private University Ethics Committee). Participation was anonymous and voluntary and all participants provided informed consent.

## Consent

The authors have nothing to report.

## Conflict of Interests

The authors declare no conflicts of interest.

## Transparency Statement

The lead author, Sebastian Fitzek, affirms that this manuscript is an honest, accurate, and transparent account of the study being reported; that no important aspects of the study have been omitted; and that any discrepancies from the study as planned (and, if relevant, registered) have been explained.

## Supporting information

GRIPP2 SF Checklist HScR.

STROBE Checklist CrossSectional.

**Supplementary Table S1:** Ordered logistic regression models of patient‐reported outcomes by disclosure (OR, 95% CI).

## Data Availability

All cited references were screened for retractions and corrections using Retraction Watch and PubMed; none were identified. Data and analysis code are available from the corresponding author upon reasonable request.
