# Supplementary material for: Asexuality Disclosure in Healthcare: Attachment and Patient‐Reported Experiences in a Cross‐Sectional Pilot Survey
Source: Health Sci Rep. 2026 Mar 11;9(3):e72094. doi: 10.1002/hsr2.72094 (PMC13098086; doi:10.1002/hsr2.72094)
Supplement: Supplementary file 1 — GRIPP2 SF Checklist HScR. [file HSR2-9-e72094-s002.docx]

# ​​GRIPP2–SF Checklist — for “Asexuality disclosure in healthcare: attachment and patient‑reported experiences in a pilot survey”

Study design: Cross-sectional online pilot survey of self‑identified asexual adults (N=47). See Main Manuscript — Methods: “Patient and Public Involvement (GRIPP2‑SF)”.

**1. Aim of PPI**

To ensure respectful, non-pathologising language and acceptable survey wording; to improve the clarity and inclusivity of items (especially microlabels and clinical scenarios); and to develop an accessible lay summary for dissemination.

**2. Methods for PPI**

We consulted community members ( advisors) before data collection. They reviewed recruitment messages, piloted the questionnaire for clarity and burden, and commented on lay summary plans. PPI contributors were not study participants and did not participate in data analysis or authorship decisions.

**3. Results of PPI**

PPI led to: (a) adoption of non‑pathologising terminology, (b) inclusion of “asexual” and microlabel options, (c) clearer item phrasing around disclosure contexts, and (d) a plan for a lay summary. No adverse effects were reported, and the survey length remained acceptable.

**4. Influence of PPI on the study**

PPI influenced instrument content and tone (neutral prompts and inclusive labels) and strengthened the patient-centred framing of implications for practice. The analytical decisions and authorship were not influenced by PPI.

**5. Reflections/critical perspective**

Strengths: Early consultation improved face validity and acceptability; contributors highlighted the non-pathologising language. Limitations: There were no formal co-researcher roles, limited diversity of contributors, and no dedicated PPI budget. Future work should include trained community co‑researchers, provide reimbursement, and involve PPI across analysis/interpretation stages.

Where reported in manuscript: Methods — “Patient and Public Involvement (GRIPP2‑SF)”; Discussion/Implications.
