# Supplementary material for: Asexuality Disclosure in Healthcare: Attachment and Patient‐Reported Experiences in a Cross‐Sectional Pilot Survey
Source: Health Sci Rep. 2026 Mar 11;9(3):e72094. doi: 10.1002/hsr2.72094 (PMC13098086; doi:10.1002/hsr2.72094)
Supplement: Supplementary file 2 — STROBE Checklist CrossSectional. [file HSR2-9-e72094-s001.docx]

# STROBE Checklist for Cross-Sectional Studies

Manuscript: “Asexuality disclosure in healthcare: attachment and patient-reported experiences in a pilot survey”.

| Item | Recommendation | Where reported | Notes |
| --- | --- | --- | --- |
| 1 | Title and abstract | Title; Abstract | Design identified as cross-sectional; key outcomes and covariates summarised. |
| 2 | Background/rationale | Introduction — Background | Motivation: misunderstanding/stigma in asexual patients; gap on disclosure & attachment. |
| 3 | Objectives | Introduction — Objectives | Describe ECR-R levels and test association of disclosure with outcomes. |
| 4 | Study design | Methods — Design | Cross-sectional online survey; convenience sampling. |
| 5 | Setting | Methods — Setting and participants | Online recruitment via community/university channels; timeframe as per manuscript. |
| 6 | Participants | Methods — Participants | Eligibility: self-identified asexual adults; final N=47; recruitment/consent online. |
| 7 | Variables | Methods — Measures/Outcomes | Disclosure (no/depends/yes), misunderstanding, stigma, discomfort; covariates: age, ECR-R Anxiety/Avoidance. |
| 8 | Data sources/measurement | Methods — Measures | ECR-R validated scales; composite indices with reliability (α). |
| 9 | Bias | Methods — Limitations; Discussion | Self-selection, self-report, cross-sectional design; small N. |
| 10 | Study size | Methods — Participants | Convenience sample; N determined pragmatically (pilot). |
| 11 | Quantitative variables | Methods — Statistical analysis | Outcomes treated as continuous (OLS) with ordinal sensitivity (ordered logit). |
| 12 | Statistical methods | Methods — Statistical analysis | OLS with HC3 robust SEs; ordered logit; FDR for multiplicity; bootstrap CIs for g; listwise deletion; marginal means. |
| 13 | Participants (flow) | Results — Sample characteristics; Table 1 | Usable responses (N=47) described; attrition noted qualitatively. |
| 14 | Descriptive data | Results — Table 1; Figures 1–3 | Age, gender identity; ECR-R means/SD; reliability α. |
| 15 | Outcome data | Results — Table 3; Figures 4–5; Supplement S1 | Associations between disclosure and outcomes; marginal means; ordered-logit ORs. |
| 16 | Main results | Results — Table 3; Discussion — Principal findings | Selective disclosure associated with more misunderstanding/stigma; consistent disclosure with less discomfort (adjusted, FDR-robust). |
| 17 | Other analyses | Results — Supplement S1 | Ordered-logit sensitivity analyses reported as ORs with 95% CI. |
| 18 | Key results | Discussion — Principal findings | Summarises adjusted associations and relative role of attachment. |
| 19 | Limitations | Discussion — Strengths and limitations | Small convenience sample; cross-sectional; self-report; generalisability caveats. |
| 20 | Interpretation | Discussion — Interpretation and implications | Aligns with prior literature; practical recommendations provided. |
| 21 | Generalisability | Discussion — Limitations | Generalisability limited beyond similar contexts; calls for multisite studies. |
| 22 | Funding | Declarations — Funding | No specific grant; COI declared; ethics and consent reported. |
