# Supplementary material for: Asexuality Disclosure in Healthcare: Attachment and Patient‐Reported Experiences in a Cross‐Sectional Pilot Survey
Source: Health Sci Rep. 2026 Mar 11;9(3):e72094. doi: 10.1002/hsr2.72094 (PMC13098086; doi:10.1002/hsr2.72094)
Supplement: Supplementary file 3 — Supplementary Table S1: Ordered logistic regression models of patient‐reported outcomes by disclosure (OR, 95% CI). [file HSR2-9-e72094-s003.docx]

**Supplementary Table S1 — Ordered logistic regression models of patient-reported outcomes by disclosure (OR, 95% CI)**

Analytic N per model equalled the number of participants with non-missing data on included variables (total sample N=47); listwise deletion was used.

Note: The “yes/always” subgroup was n=4; interpret its estimates with caution due to wide CIs.

(Ordinal model cutpoints are model intercepts and are not reported as effects.)

**Misunderstood**

| **Term** | **OR (95% CI)** | **p** |
| --- | --- | --- |
| disclose_depends | 5.69 (1.45, 22.32) | 0.01 |
| disclose_yes | 9.08 (1.13, 73.19) | 0.04 |
| age | 1.02 (0.98, 1.07) | 0.36 |
| ANXIETY | 1.72 (1.00, 2.95) | 0.05 |
| AVOIDANCE | 1.17 (0.72, 1.91) | 0.52 |
| 1/2 | 37.86 (2.96, 484.43) | 0.005 |
| 2/3 | 0.68 (0.32, 1.46) | 0.33 |
| 3/4 | 1.13 (0.62, 2.07) | 0.68 |
| 4/5 | 0.94 (0.44, 1.99) | 0.87 |

**Stigma**

| **Term** | **OR (95% CI)** | **p** |
| --- | --- | --- |
| disclose_depends | 5.32 (1.30, 21.80) | 0.02 |
| disclose_yes | 3.39 (0.31, 36.74) | 0.32 |
| age | 1.01 (0.96, 1.06) | 0.62 |
| ANXIETY | 1.46 (0.86, 2.49) | 0.17 |
| AVOIDANCE | 1.15 (0.69, 1.91) | 0.58 |
| 1/2 | 24.46 (1.78, 336.25) | 0.02 |
| 2/3 | 0.73 (0.36, 1.46) | 0.37 |
| 3/4 | 0.75 (0.35, 1.60) | 0.46 |
| 4/5 | 0.70 (0.27, 1.78) | 0.45 |

**Uncomfortable**

| **Term** | **OR (95% CI)** | **p** |
| --- | --- | --- |
| disclose_depends | 1.05 (0.32, 3.41) | 0.93 |
| disclose_yes | 0.05 (0.00, 0.68) | 0.02 |
| age | 1.03 (0.99, 1.09) | 0.17 |
| ANXIETY | 0.92 (0.56, 1.49) | 0.73 |
| AVOIDANCE | 1.28 (0.83, 1.97) | 0.27 |
| 1/2 | 1.21 (0.13, 11.23) | 0.87 |
| 2/3 | 1.20 (0.70, 2.07) | 0.51 |
| 3/4 | 0.90 (0.49, 1.64) | 0.73 |
| 4/5 | 1.45 (0.81, 2.61) | 0.21 |

Cutpoints (log-odds scale), not interpretable as predictors’ effects:

κ1 (between categories 1|2) = β1; κ2 (2|3) = β2; κ3 (3|4) = β3; κ4 (4|5) = β4.
